# Remarks on Pellagra

**Published:** 1846-01

**Authors:** Meredith Clymer

**Affiliations:** Physician to the Philadelphia Hospital, &c. &c.; 230 Spruce St.


*Remarks on Pellagra.* By MEREDITH CLYMER, M. D., Physician to the Philadelphia Hospital, &c. &c.

For a long while this fearful malady was supposed to be endemic to the Lombardo-Venitian provinces, and was known as *Scorbutus Alpinus, Lepra Lombardica.* In 1818, it was observed for the first time in the Canton of La Teste, in Gascony, France; and since then it has been found to prevail to a greater or less degree in the Gironde. Several cases, within the last few years, have been observed at Paris, where the disease passed for some time unrecognized even by Biett, and subsequently by Cazenave.* Recent communications made to the French Minister of Agriculture, and the Academy of Medicine, show that it prevails to a great extent in the *landes*† of the Gironde. It

*A communication on these cases was made to the writer from Paris, and published in the 6th Vol. of this Journal, p. 226.

† The "Landes" are vast sandy flats, interspersed with patches of pasture, and bounded towards the sea by a range of sandy downs, with an adjacent line of successive lagoons They occupy four-fifths of the department of that name, and all the western portion of the Gironde. Their surface is arid, except for four months in the year, when the rains form extensive pools in the depressed portions. The lower classes are in a most degraded state, both mentally and physically, subsisting chiefly on maize and millet porridge.

is known now that it has long been endemic in the provinces of Asturias, in Spain. The best and clearest description of this malady has been given by Dr. Leon Marchand, Secretary of the Board of Health of the Gironde, who has been engaged in in its study since 1836.

The most prominent external character is a squamous erythema, occupying all the exposed portions of the body, though chiefly the dorsal surface of the hands; disappearing towards autumn, and leaving on the skin shining cicatrices, resembling those from a burn; and returning annually each spring, with a train of symptoms, whose intensity is in proportion to the duration of the disease. The eruption may assume successively the papular, vesicular and pustular forms. The general phenomena accompanying the local affection diminish with it, to be reproduced, in an aggravated form, each successive spring; and finally to become persistive, under the continued influence of the causes, until the disease attains a degree of gravity which renders it necessarily mortal. The most constant constitutional symptoms are a scorbutic condition of the mouth and gums; dyspepsia; diarrhœa and vomiting; pains and debility in the limbs; unsteadiness in the gait; dulness of the senses and intellect, terminating in violent mania, or dementia, with a disposition to suicide by drowning.

It is hereditary; Dr. Marchand has seen five generations pellagrous.

So far no light has been thrown on the nature of the disease by pathological anatomy.

Pellagra occurs in sterile countries, and low marshy districts, in the midst of depressing and debilitating influences. According to Jolly,*—and most authors agree with him,—the infected emanations from marshes and lagoons; insalubrious dwellings; insufficient clothing; filth,—all the circumstances of extreme misery—are the conditions which contribute to the development of this disease, called by many writers, for this reason, *mal de misére.* That this cannot be the only, or, indeed, efficient cause, appears from the fact of its not occurring indiscriminately where there is a great amount of individual wretchedness. Some of the Italian physicians—and Gibert amongst the French—think it due to exposure to the sun's influence—hence it has been called *Insolazione de Primavera.* But the disease does not date back earlier than the beginning of the last century, and does not prevail in tropical countries.

In a thesis on this obscure and interesting affection, lately sustained before the Faculty of Medicine of Paris, by Dr. Theophile

* Report made to the Academy of Medicine, June 3d, 1845.

ROUSSEL, a highly ingenious and important view has been taken of the efficient cause of Pellagra. " Wherever Pellagra is observed, you find," says Dr. ROUSSEL, "a class of persons subsisting, nearly exclusively, for a part of the year at least, on Indian corn, either alone, or associated with some analogous cereal." Topographical details show that every where in Europe, where the culture of Indian corn prevails, Pellagra is found. Dr. ROUSSEL asserts, from observation and inquiry, that in the departments in France, where Indian corn is not cultivated, there is no Pellagra, although the misery of the peasants is great. Pellagra too does not seem to extend beyond the latitude where the culture of Indian corn stops.

The chronological history of the cultivation of this cereal in Europe, furnishes another argument in support of this proposition. In the northern provinces of Spain, the cultivation of Indian corn did not become of importance till the end of the sixteenth or commencement of the seventeenth century. Spain is the only country in which Pellagra was met with as early as the first half of the seventeenth century. In Italy the correlativeness of the extension of the culture of maize, and the appearance of Pellagra, is established on precise dates, and proved by numerous undoubted authorities. It was at the close of the seventeenth century, and the beginning of the eighteenth, that Indian corn was cultivated to any great extent in northern Italy, so as to predominate over other cereals; and it was about 1750, that the Italian physicians first met with Pellagra. In France, its culture is of still more recent date ; and it was not till 1818, that it was known in that country. It is worthy of notice that Indian corn, of all the cereals, is that which furnishes less azote, and has the harvests most frequently damaged. Pellagra has never been observed beyond 35° or 37° and 46°, which exactly enclose the zone where Indian corn is cultivated.

From these facts, the proposition of Dr. Roussel would seem to be established—the predominance of the cultivation of Indian corn, and the nearly exclusive alimentation with it, with the development of Pellagra.

This idea is by no means novel, although it has never been presented in so complete a manner before. THOUVENAL announced the fact in 1798, in his *Traité du Climat d'Italie.* He stated that maize constituted the chief food of pellagrous persons, and he attempted to show the striking relation between the epoch of the extended culture of Indian corn in Italy, and the period of the prevalence of Pellagra. Dr. Roussel, quotes from Dr. Mazzari, who, after a long examination of the question, concludes that it is exclusive vegetable nourishment, during winter and spring, which engenders Pellagra; and adds that this vegetable diet consists of Indian corn, in the form of *polenta** in the Venetian, and bread in the Lombardian provinces. At the last meeting of the Scientific Congress of Italy, held at Milan, Dr. Ballardini stated, that Pellagra had followed the introduction of Indian corn, and that its course could be traced by pursuing the progress of that cereal. He demonstrated that those regions which were under the same influences to which Pellagra was commonly attributed, and where Indian corn was not grown, were totally exempt from that disease. This correlative influence is so well established in the minds of some of the Italian physicians, that they have named the disease *Raphania Maïztica.*

230 *Spruce St., Dec.* 15th, 1845.

* A baked pudding of corn meal.

